# Changes in Acetyl CoA Levels during the Early Embryonic Development of *Xenopus laevis*

**DOI:** 10.1371/journal.pone.0097693

**Published:** 2014-05-15

**Authors:** Yugo Tsuchiya, Uyen Pham, Wanzhou Hu, Shin-ichi Ohnuma, Ivan Gout

**Affiliations:** 1 Institute of Structural and Molecular Biology, University College London, London, United Kingdom; 2 UCL Institute of Ophthalmology, University College London, London, United Kingdom; Radboud University Nijmegen, Netherlands

## Abstract

Coenzyme A (CoA) is a ubiquitous and fundamental intracellular cofactor. CoA acts as a carrier of metabolically important carboxylic acids in the form of CoA thioesters and is an obligatory component of a multitude of catabolic and anabolic reactions. Acetyl CoA is a CoA thioester derived from catabolism of all major carbon fuels. This metabolite is at a metabolic crossroads, either being further metabolised as an energy source or used as a building block for biosynthesis of lipids and cholesterol. In addition, acetyl CoA serves as the acetyl donor in protein acetylation reactions, linking metabolism to protein post-translational modifications. Recent studies in yeast and cultured mammalian cells have suggested that the intracellular level of acetyl CoA may play a role in the regulation of cell growth, proliferation and apoptosis, by affecting protein acetylation reactions. Yet, how the levels of this metabolite change *in vivo* during the development of a vertebrate is not known. We measured levels of acetyl CoA, free CoA and total short chain CoA esters during the early embryonic development of *Xenopus laevis* using HPLC. Acetyl CoA and total short chain CoA esters start to increase around midblastula transition (MBT) and continue to increase through stages of gastrulation, neurulation and early organogenesis. Pre-MBT embryos contain more free CoA relative to acetyl CoA but there is a shift in the ratio of acetyl CoA to CoA after MBT, suggesting a metabolic transition that results in net accumulation of acetyl CoA. At the whole-embryo level, there is an apparent correlation between the levels of acetyl CoA and levels of acetylation of a number of proteins including histones H3 and H2B. This suggests the level of acetyl CoA may be a factor, which determines the degree of acetylation of these proteins, hence may play a role in the regulation of embryogenesis.

## Introduction

Vast numbers of enzyme-catalysed biochemical transformations are dependent on cofactors, which are non-protein, chemical compounds that associate with enzymes and assist their biological activity. Coenzyme A (CoA) is an essential and ubiquitous cofactor made from pantothenate (vitamin B5), ATP, and cysteine [1]. CoA acts as a carrier of acyl groups and transports biologically active carboxylic acids, including small organic acids and fatty acids, between different enzymatic reactions in the form of CoA thioesters. CoA thioesters are important intermediates and precursors in numerous metabolic pathways, including oxidation of glucose and fatty acids and biosynthesis of lipids. Acetyl CoA is a CoA thioester which is centrally placed at a junction of multiple catabolic and anabolic pathways. Mitochondrial acetyl CoA, derived from catabolism of glucose and beta-oxidation of fatty acids, can be further oxidised in the citric acid cycle for energy production, while cytosolic acetyl CoA is a precursor for lipid and cholesterol biosynthesis. Additionally, both mitochondrial and nucleocytoplasmic acetyl CoA serve as co-substrates for protein acetylation reactions, linking cellular metabolism to protein post-translational modifications.

Cellular levels of CoA and CoA thiosters are not constant and fluctuate significantly under conditions such as fasting/feeding, in response to nutrients and hormones and during energetic stress and cell growth [2]–[8]. Such changes in CoA metabolites not only reflect a shift in the metabolic activity of a cell in response to different intracellular and extracellular stimuli, but can themselves act as a signal for regulating cellular processes [8]–[10]. Notably, recent accumulating evidence suggests that cellular levels of acetyl CoA can directly influence cell growth, cell cycle, differentiation and apoptosis by affecting protein acetylation reactions and epigenetic modifications [8], [11]–[13].

Three forms of protein acetylation have been identified to date: O-linked, N^ε^-linked, and N^α^-linked acetylation. In all three types of acetylation reactions acetyl CoA donates the acetyl group to the acceptor protein, releasing free CoA. N^ε^-linked acetylation of histones and transcription factors has been recognised for many years as a post-translation modification important for regulation of gene transcription [14], [15]. It is generally accepted that this type of acetylation is dynamically regulated by a balance between histone acetyl transferases (HATs) and histone deacetylases (HDACs), which themselves are regulated by gene expression and post-translational modifications, such as phosphorylation and acetylation [16], [17]. However, a number of recent studies have suggested that the level or availability of acetyl CoA is also an important factor influencing acetylation reactions [18]–[20]. In cultured mammalian cells disruption of ATP citrate lyase (ACL), an enzyme that supplies nucleocytoplasmic acetyl CoA, caused a reduction in histone acetylation and induced differentiation in mouse C2C12 myoblasts [12], [21]. Conversely, reduced expression of acetyl CoA carboxylase 1 (ACC1), a cytosolic enzyme that competes with HATs for nucleocytoplasmic acetyl CoA, caused an increase in bulk histone acetylation [22]. Moreover, a surge in intracellular acetyl CoA during the oxidative phase of yeast metabolic cycles induced the Gcn5p/SAGA-catalysed acetylation of histones at growth genes and acted as a trigger for initiating a cellular growth programme and cell cycle entry [8], [11]. Intracellular acetyl CoA levels have also been implicated in regulation of N^α^-linked acetylation and apoptosis. Overexpression of Bcl-xL in human cells caused a reduction in cellular acetyl CoA and a concomitant decrease in protein N^α^-acetylation, which could be restored by increasing acetyl CoA levels by addition of citrate or acetate [13]. High levels of acetyl CoA and N^α^-acetylation were shown to be associated with increased susceptibility to apoptotic stimuli [13].

Considering the emerging role of intracellular acetyl CoA levels in the regulation of cell growth, differentiation, cell cycle, and apoptosis, we sought to measure changes in the level of this metabolite *in vivo* during the embryonic development of a vertebrate. Most *in vivo* measurements of CoA species have been limited to tissues of adult organisms subjected to different conditions (such as feeding and fasting), whereas how the levels of CoA species change in a developing organism is largely unknown. In this study we used embryos of *Xenopus laevis*, a widely used model species for studying cellular processes underlying embryogenesis. We report here that levels of acetyl CoA *in vivo* change dramatically during early embryonic development. We observed that changes in acetyl CoA appear to correlate with changes in the whole-embryo levels of N^ε^-acetylation of a number of proteins, including histones H2B and H3.

## Materials and Methods

### Ethics Statement

All experiments involving *Xenopus laevis* were performed under a Home Office Project Licence in accordance with the Animals (Scientific Procedures) Act 1986.

### Chemicals and antibodies

CoA standards and all common chemicals were from Sigma-Aldrich. All antibodies were from Cell Signaling Technologies. The following antibodies were used: acetyl-histone H2A (Lys 5) (#2576); acetyl-histone H2B (Lys 5) (#2574); acetyl-histone H3 (Lys 9) (#9649); acetyl histone H3 (Lys 18) (#9675); acetyl-histone H4 (Lys 8) (#2594); acetylated-lysine antibody (#9441); histone H2A (#2578); histone H2B (#8135); histone H3 (#4499); histone H4 (#2935).

### *In vitro* fertilisation of *Xenopus* eggs and sample collection

*In vitro* fertilisation was performed as previously described [23]. Fertilised eggs were kept in 0.1X Modified Barth's Solution (MBS) until the appropriate stages were reached for collection. Embryonic stages were determined according to Nieuwkoop and Faber [24]. 30 and 20 embryos were collected per stage for HPLC and Western blotting, respectively. Embryos were transferred to 1.5 ml tubes and the incubation medium was removed before snap-freezing in dry ice. Embryos were stored at −80°C until use.

### Preparation of samples for HPLC analysis

Frozen embryos (30 embryos per each stage) were partially thawed on ice and 150 µl of ice cold 5% perchloric acid (PCA) was immediately added. Samples were homogenised using a pipette, briefly vortexed and blended with a tissue tearer before centrifugation at 18,000 g for 5 minutes at 4°C. The supernatant, which contains CoASH and short-chain CoA esters, was collected and 1 M triethanolamine was added to a final concentration of 100 mM. The pH was adjusted to pH 6 with 5 M K_2_CO_3_ and potassium perchlorate pellet was removed by centrifugation at 18,000 g for 3 minutes at 4°C. Injection mixture was made by adding 10 µl of 1.5 M Na_2_H_2_PO_4_, 10 µl of 100 mM Tris-(2-carboxyethyl) phosphine hydrochloride (TCEP), 5 µl of 100 mM ethylenediaminetetraacetic acid (EDTA), and 9 µl of methanol to 66 µl of neutralised PCA extract. The injection mixture was filtered through a 0.2 µm PVDF filter and 50 µl was loaded onto the HPLC column. For analysis of total short chain CoA esters, 5 M KOH and 100 mM TCEP were added to neutralised PCA extracts to final concentrations of 0.5 M and 10 mM, respectively. After incubation at room temperature for 10 minutes, the pH was adjusted to pH 6 with 2 M HCl and injection mixture was prepared as above.

### HPLC analysis of CoA compounds

This is a modification of a previously published method [25]. The column used was a Kinetex C18 column (100 X 4.60 mm) with 2.6 µm particle size and 100 Å pore size (Phenomenex). The column temperature was kept at 40°C by a water bath. Solvent A consisted of 150 mM Na_2_H_2_PO_4_ and 9% methanol and solvent B consisted of 150 mM Na_2_H_2_PO_4_ and 30% methanol. CoA compounds were eluted isocratically with solvent A at a flow rate of 0.8 ml/min for the first 20 minutes after which a linear gradient to 100% solvent B was applied over 5 min at a flow rate of 0.5 ml/min. This was followed by a linear gradient back to 100% solvent A and the flow rate was returned to 0.8 ml/min over 5 minutes. The column was re-equilibrated with 100% solvent A for 10 minutes before the start of the next run. Elution of CoA compounds was monitored by absorbance at 254 nm. Peaks for CoA compounds in *Xenopus* extracts were identified by comparison of retention times with those of authentic standards determined on the same day. The retention time for each compound and day-to-day variability in retention times are shown in Table S1. The position of the CoASH and acetyl CoA peaks were also confirmed using internal standards (Figure S1). The retention times of CoASH and acetyl CoA peaks were not affected by the PCA extract (Figure S1). Approximately 90% of the peak areas of authentic CoASH and acetyl CoA standards added to a *Xenopus* PCA extract, in quantities similar to endogenous levels of CoASH and acetyl CoA, could be recovered (Figure S1, also see Figure S2). Therefore, CoA compounds in *Xenopus* extracts were quantified by comparison of peak areas with those of external standards as this can give reasonable estimation of true levels of CoASH and acetyl CoA in *Xenopus* extracts. Peak area was quantified by Borwin chromatography software.

### Western blotting

Frozen embryos (20 embryos per each stage) were partially thawed on ice and 80 µl of ice-cold Lysis buffer (50 mM Tris/HCl pH 7.5, 150 mM NaCl, 50 mM NaF, 5 mM Na_4_P_2_O_7_, 1 mM EDTA, 10% glycerol, 1% Triton X100 and protease inhibitor cocktail (Roche)) was added. Samples were homogenised using a pipette, briefly vortexed and blended with a tissue tearer before centrifugation at 18,000 g for 10 minutes at 4°C. The supernatant was carefully transferred, avoiding the fat layer on the surface, to a fresh tube and centrifuged again as above for 5 minutes. Approximately 40 µg of soluble protein in the supernatant was heated in SDS loading buffer at 99°C for 10 minutes, separated by SDS-PAGE and transferred to a PVDF membrane (BioRad). The membrane was blocked with 5% milk powder in Tris-buffered saline containing 0.1% Tween 20 (TBSt) and incubated overnight at 4°C with anti-acetyl lysine, anti-acetyl histone or anti-total histone antibodies diluted (1/1000) in TBSt. Bands were visualised by enhanced chemiluminescence with HRP-conjugated secondary antibodies (Promega). The size and intensity of bands were quantified using MultiGauge software (Fujifilm).

### Microinjection

Embryos were dejellied 30–45 min after fertilisation with 2% cysteine in water and washed with 0.1X MBS. Embryos were transferred to 0.2X MBS containing 4% Ficoll and 50 µg/ml gentamycin and injected with one, two or three pulses (approximately 10 nl each) of 1 mM acetyl CoA in water. Control embryos were injected with water. Injected embryos were allowed to progress to stage 3 (approximately 3 hours) at 18°C. 10 injected embryos were combined and collected as above for HPLC or Western blot analysis.

### Statistical analysis

Where appropriate, values are given as means ± SEM and statistical significance was calculated using Student's *t* test for unpaired samples. Statistical significance was taken as p<0.05.

## Results

### Detection of CoA species in *Xenopus* embryos by HPLC

**Figure 1:**
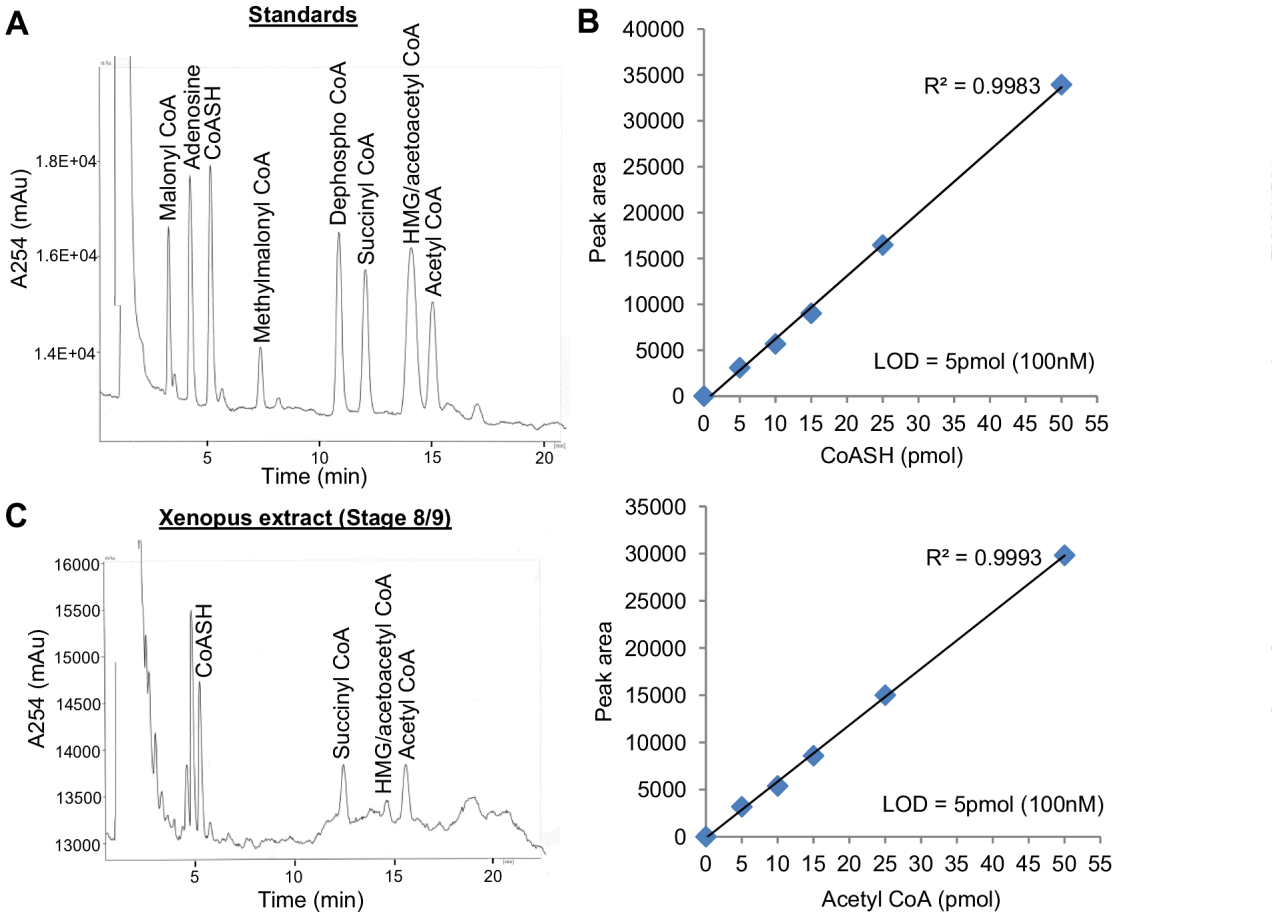
Chromatographic separation of CoA standards and a PCA extract of *Xenopus* embryos. (A) HPLC chromatogram illustrating separation of CoA compounds. Standards of CoASH, CoA thioesters and adenosine (20–50 pmol each) were separated on a C18 column (Kinetex C18 100 X 4.60 mm column with 2.6 µm particle size and 100 Å pore size) using a mobile phase consisting of 150 mM Na_2_H_2_PO_4_ and 9% methanol and a flow rate of 0.8 ml/min (see Materials and Methods for details). Standards were made up in mobile phase, which additionally contained 5 mM EDTA and 10 mM TCEP before injection. CoA compounds were detected by absorbance at 254 nm. Retention times (in minutes): malonyl CoA, 3.21; adenosine, 4.16; CoASH, 5.06; methylmalonyl CoA, 7.26; dephospho CoA, 10.7; succinyl CoA, 11.87; HMG/acetoacetyl CoA, 13.96; acetyl CoA, 14.83. See Table S1 for day-to-day variability in retention times. (B) Graphs showing linearity between the peak area and the amount of CoASH and acetyl CoA injected. Different amounts of CoASH and acetyl CoA standards were injected and the peak areas were determined by Borwin chromatography software. Each data point represents the mean of duplicate measurements. The limit of detection (LOD) for each compound was determined to be 5 pmol (100 nM). (C) Representative chromatogram showing separation of a PCA extract of stage 8/9 *Xenopus* embryos. CoA peaks were identified by comparison of retention times with those of authentic standards determined on the same day. Peaks corresponding to CoASH, succinyl CoA, HMG/acetoacetyl CoA and acetyl CoA were detected. Retention times (in minutes): CoASH, 5.21; succinyl CoA, 12.32; HMG/acetoacetyl CoA, 14.44; acetyl CoA, 15.43.

**Figure 2:**
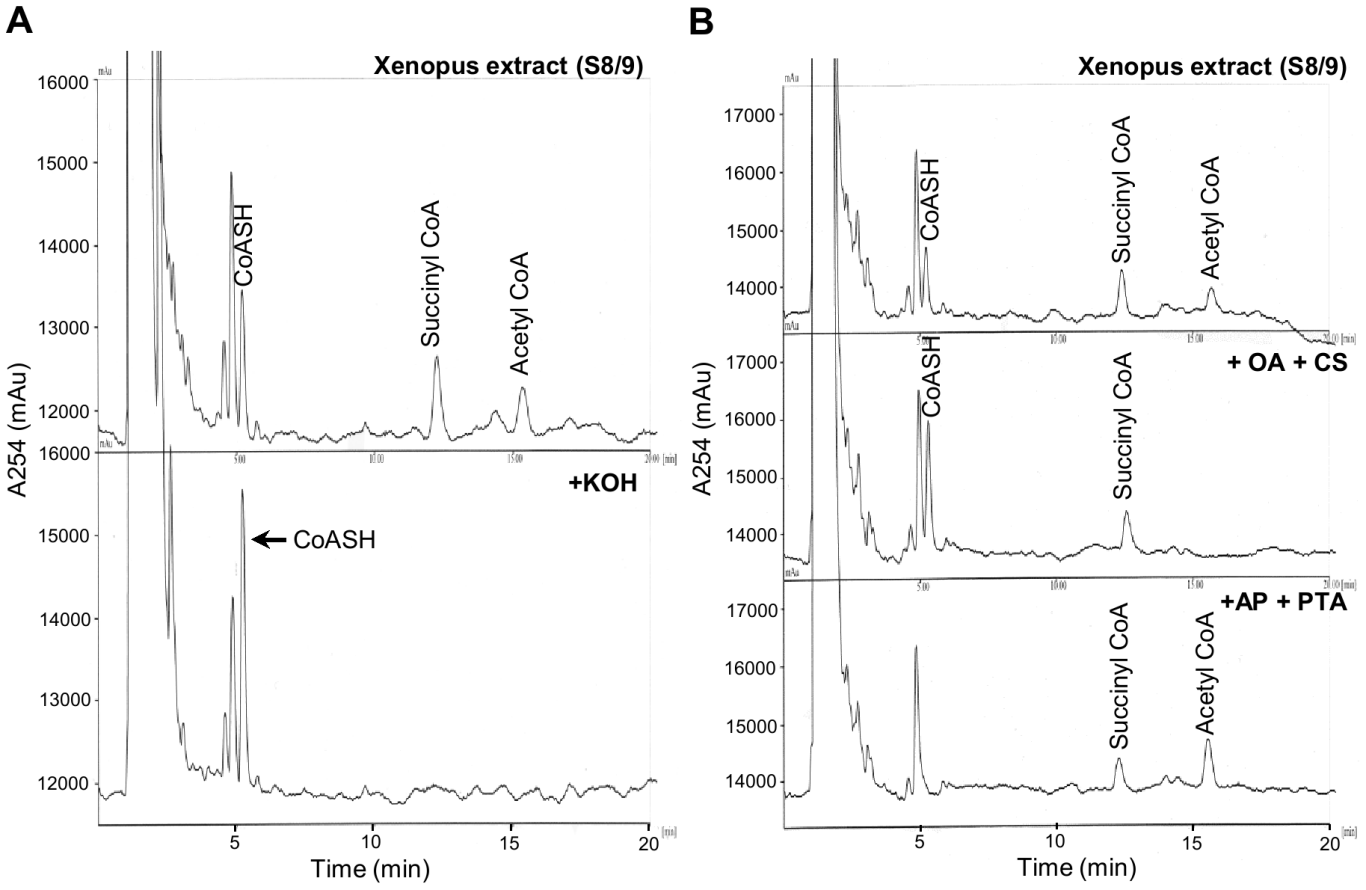
Validation of the identity and purity of CoASH and acetyl CoA peaks detected in *Xenopus* embryo extracts. (A) 30 stage 8/9 embryos were extracted with 150 µl 5% PCA and the PCA soluble fraction was neutralised with TEA/K_2_CO_3_. CoA compounds in neutralised PCA extracts were analysed by HPLC before and after KOH treatment as described in the Materials and Methods section. KOH treatment caused conversion of peaks corresponding to succinyl CoA and acetyl CoA, to free CoASH. Retention times (in minutes): CoASH, 5.15; succinyl CoA, 12.13; acetyl CoA, 15.17. (B) 30 stage 8/9 embryos were extracted with 150 µl 5% PCA and the PCA soluble fraction was neutralised with TEA/K_2_CO_3_. The neutralised extract was analysed by HPLC without further treatment (top panel), or after incubation for 10 min at 30°C with 0.11 mM oxaloacetate (OA) and citrate synthase (CS) (middel panel), or with 60 mM KCl, 9.4 mM acetyl phosphate (AP), and phosphotransacetylase (PTA) (bottom panel). Incubation of *Xenopus* extract with citrate synthase, in the presence of oxaloacetate, caused complete disappearance of the peak corresponding to acetyl CoA and an increase in the size of the peak corresponding to CoASH (peak areas before treatment: CoASH, 9337.25; succinyl CoA, 11626; acetyl CoA, 6678. Peak areas after treatment: CoASH, 18217.5; succinyl CoA, 11252.25; acetyl CoA, no peak detected). Conversely, incubation with phosphotransacetylase, in the presence of acetyl phosphate, completely removed the peak corresponding to CoASH and increased the size of the acetyl CoA peak (peak areas after treatment: CoASH, not detected; succinyl CoA, 9278.5; acetyl CoA, 15213.5). These results confirm unequivocally the identity of the CoASH and acetyl CoA peaks and that there are no other compounds co-eluting with these peaks that absorb at 254 nm. Retention times in minutes: top panel; CoASH, 5.17; succinyl CoA, 12.26; acetyl CoA, 15.51; middle panel; CoASH, 5.24; succinyl CoA, 12.43; acetyl CoA, not detected; bottom panel; CoASH, not detected; succinyl CoA, 12.12; acetyl CoA, 15.36.

Using the HPLC conditions described in the Materials and Methods section, we were able to separate and detect peaks for commercially obtained standards of CoASH and a number of CoA thioesters (Figure 1A). The lower limit of detection for CoASH and acetyl CoA was determined to be 5 pmol (Figure 1B). HPLC analysis of PCA extracts of *Xenopus* embryos detected peaks corresponding to CoASH, succinyl-, HMG/acetoacetyl- and acetyl-CoA (Figure 1C). The malonyl CoA and HMG/acetoacetyl CoA peaks were not consistently detectable due to their small size and/or the presence of other closely eluting peaks. Since our detection method relies on UV absorption at 254 nm, which cannot specifically identify CoA compounds, preliminary experiments were performed to check the identity and purity of the CoA peaks observed in *Xenopus* PCA extracts. CoA thioesters are readily hydrolysed into free CoASH and corresponding carboxylic acids under alkaline conditions. As can be seen in Figure 2A, treatment of *Xenopus* PCA extracts with KOH caused disappearance of the peaks corresponding to succinyl- and acetyl CoA, and an increase in the peak corresponding to free CoASH, consistent with hydrolysis of the CoA thioesters into unesterified CoASH. The identity and purity of CoASH and acetyl CoA peaks were further verified using citrate synthase and phosphotransacetylase (Figure 2B). Incubation of a *Xenopus* extract with citrate synthase, in the presence of oxaloacetate, caused complete disappearance of the peak corresponding to acetyl CoA and an increase in the size of the peak corresponding to CoASH. Conversely, incubation with phosphotransacetylase, in the presence of acetyl phosphate, completely removed the peak corresponding to CoASH and increased the size of the acetyl CoA peak. These results confirm unequivocally the identity of the CoASH and acetyl CoA peaks and that there are no other compounds co-eluting with these peaks that absorb at 254 nm. To ensure our PCA extraction and sample preparation conditions did not cause degradation of CoA compounds or conversion of CoA esters to CoASH, we checked the recovery of known amounts of CoASH and acetyl CoA standards added at the time of PCA extraction of *Xenopus* embryos. Over 90% of added CoASH and acetyl CoA could be recovered (CoASH, 91%; acetyl CoA, 113% (n = 2)), indicating that the bulk of CoASH and acetyl CoA originally present in *Xenopus* embryos was not lost during the sample preparation procedure. An example of the recovery experiment is shown in Figure S2.

### Levels of acetyl CoA fluctuate during early *Xenopus* embryonic development

**Figure 3:**
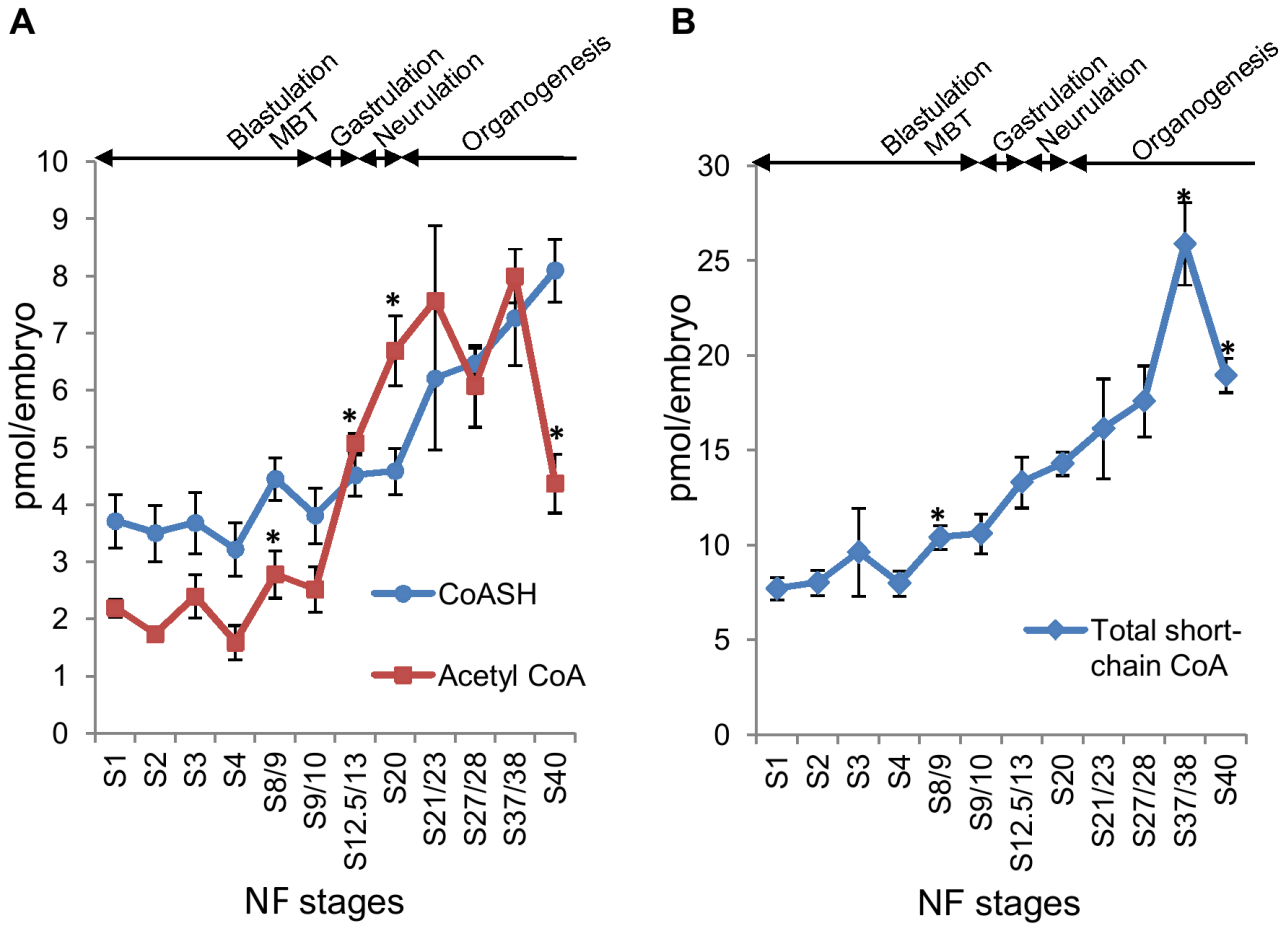
Levels of CoASH, acetyl CoA and total short-chain CoA esters during the early embryonic development of *Xenopus laevis* . (A) Levels of unesterified CoA (CoASH) and acetyl CoA were measured in neutralised PCA extracts of *Xenopus* embryos collected at different stages of early embryonic development. Embryonic stages were determined as described by Nieuwkoop and Faber [24]. 30 embryos were collected for the determination of CoA compounds at each stage. The number of independent measurements (in brackets) performed for each stage is as follows: S1 (8); S2 (5); S3 (5); S4 (9); S8/9 (6); S9/10 (9); S12.5/13 (4); S20 (6); S21/23 (3); S27/28 (3); S37/38 (4); S40 (3). Each data point represents the mean +/− SEM. * indicates statistical significance (p<0.05) compared to the immediately preceding stage as determined by Student's *t* test. (B) Levels of total acid-soluble CoA species, representing combined levels of CoASH and total short-chain CoA esters, were determined after KOH hydrolysis as described in the Materials and Methods section. The number of independent measurements performed for each stage is as follows: S1 (7); S2 (4); S3 (3); S4 (9); S8/9 (6); S9/10 (8); S12.5/13 (4); S20 (6); S21/23 (3); S27/28 (3); S37/38 (4); S40 (3). Each data point represents the mean +/− SEM. * indicates statistical significance (p<0.05) compared to the immediately preceding stage as determined by Student's *t* test.

Cells undergo dramatic biochemical and morphological changes during embryogenesis. In *Xenopus*, fertilisation of eggs is followed by a series of synchronous cleavage cycles, characterised by rapid alterations between S and M phases without gap phases and cell cycle checkpoints [26]. By the time stage 9 is reached, and the embryo is at the 4000/cell capacity, an event termed mid-blastula transition (MBT) takes place. This involves a number of important changes including loss of synchrony, appearance of gap phases, initiation of zygotic transcription and cell motility [27]. At MBT, embryos are largely composed of uncommitted sister cells [28]–[30]. During gastrulation, cells migrate and form three germ layers (the ectoderm, mesoderm and endoderm), which later differentiate into various tissues and organs during organogenesis [31]–[33]. We have measured the whole-embryo levels of free CoA, acetyl CoA and total acid-soluble CoA species, expressed as pmol per embryo, at different stages (ranging from stage 1 to stage 40) in *Xenopus* embryonic development (Figure 3). Measurement of total acid-soluble CoA species by KOH hydrolysis showed that unesterified CoASH, succinyl CoA and acetyl CoA are the predominant acid-soluble CoA species at all the stages analysed (results not shown). Levels of CoA, acetyl CoA as well as total acid soluble CoA per embryo remain relatively constant between stages 1–4, however they start to increase around stages 8–10 and continue to increase thereafter. Since embryos were grown in 0.1X MBS media in the absence of exogenous pantothenate or other precursors for CoA, the observed increase in short chain CoA esters are likely to be due to increased CoA biosynthesis from precursors stored in the yolk, decreased CoA degradation and/or increased turnover of long-chain CoA thioesters. A key finding is that there is a clear shift in the ratio of acetyl CoA/CoA during the course of early *Xenopus* embryonic development. Pre-MBT embryos contain more CoA than acetyl CoA, however after MBT CoA is overtaken by a more rapid and dramatic increase in acetyl CoA, which peaks around stage 21. This shift in the acetyl CoA/CoA ratio takes place during late gastrulation, neurulation, and early organogenesis, suggesting these processes may be associated with metabolic remodelling that results in the accumulation of acetyl CoA, either through increased production or decreased usage of acetyl CoA. At stage 40, the ratio of acetyl CoA/CoA returns to a value close to pre-MBT embryos. This coincides with stages 40/43, when lipid vesicles in cells of *Xenopus* embryos disappear and tadpoles start to feed from the environment [34].

### Changes in acetyl CoA during embryonic development correlate with global protein acetylation

**Figure 4:**
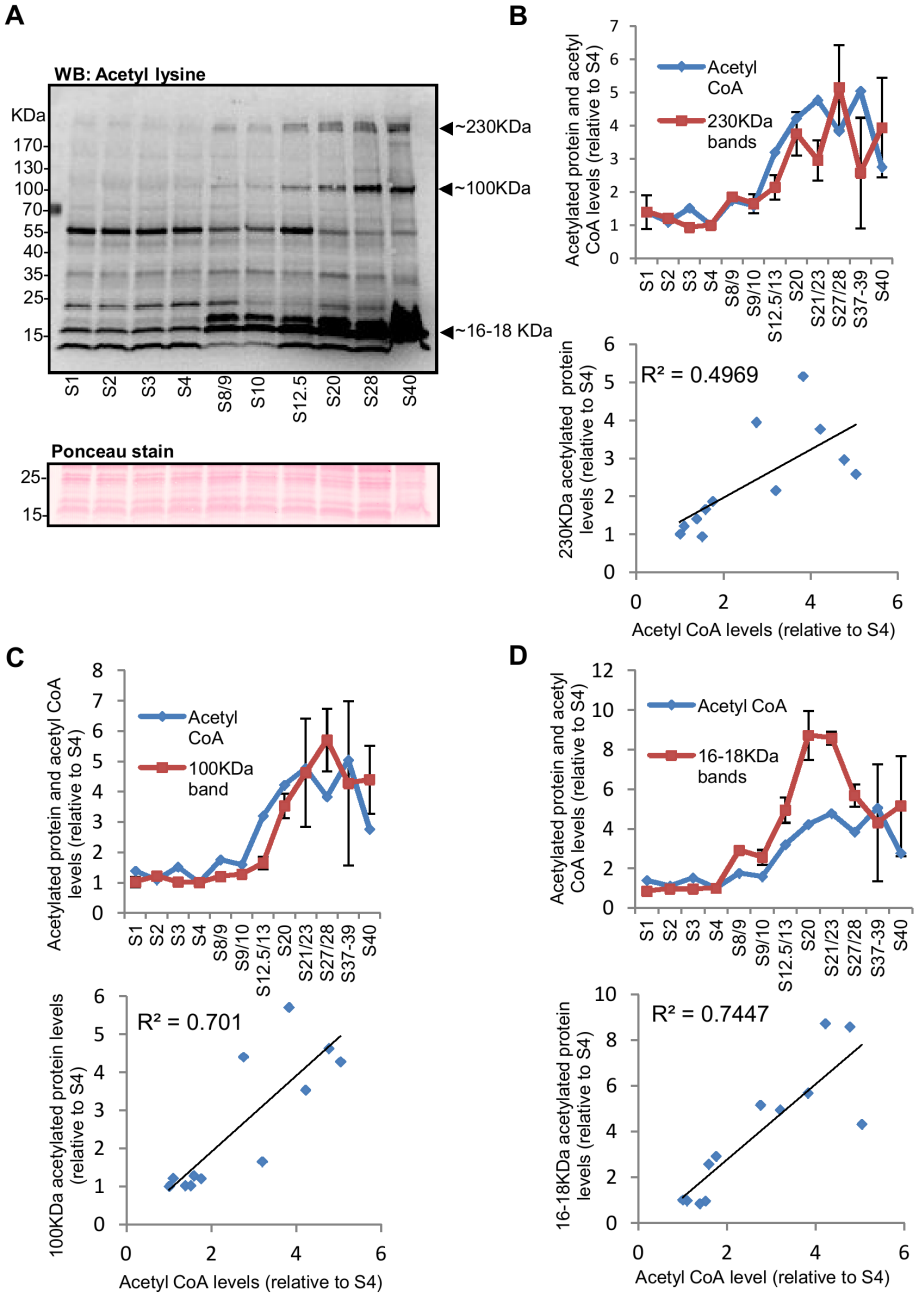
Whole-embryo acetylation levels of a number of proteins in *Xenopus* embryos appear to correlate with the pattern of change in acetyl CoA levels. (A) 20 embryos collected at different stages of development were lysed in 80 µl lysis buffer and approximately 40 µg of soluble protein was separated by SDS-PAGE, transferred to a PVDF membrane and probed with anti-acetyl lysine antibody. Equal protein loading was checked by Ponceau staining of the membrane and a Coomassie stain of a gel run in parallel. A section of the Ponceau stained membrane is shown (the complete Ponceau stained membrane and the Coomassie stained gel are shown in Figure S3). Changes in the abundance of a number of acetylated proteins, including those with molecular weights of approximately 230 KDa, 100 KDa, 16 KDa, and 18 KDa (indicated by arrows), appear to follow the overall pattern of change in acetyl CoA levels. (B–D, top graphs) Embryos at different stages of development were collected and Western blotting, with anti-acetyl lysine antibody, was performed as above. The intensity/size of acetylated protein bands at approximately 230 KDa (B), 100 KDa (C) and 16–18 KDa (D) were quantified, normalised to stage 4, and plotted against different stages of development together with levels of acetyl CoA (taken from Figure 3 and expressed relative to stage 4). Band intensity/size was normalised against stage 4 as this stage is included in all the blots performed. Each data point represents a single measurement or the mean +/− SEM of 2–7 measurements. The number of independent measurements (using samples from independent fertilisations) performed for each stage was as follows: S1 (4); S2 (4); S3 (1); S4 (7); S8/9 (1); S9/10 (7); S12.5/13 (4); S20 (5); S21 (2); S27/28 (4); S39/39 (2); S40 (3). (B–D, bottom graphs) The means of normalised band intensity/size for acetylated proteins at approximately 230 KDa (B), 100 KDa (C) and 16–18 KDa (D) were plotted against acetyl CoA levels (taken from Figure 3 and also normalised to stage 4) and R^2^ values are shown.

**Figure 5:**
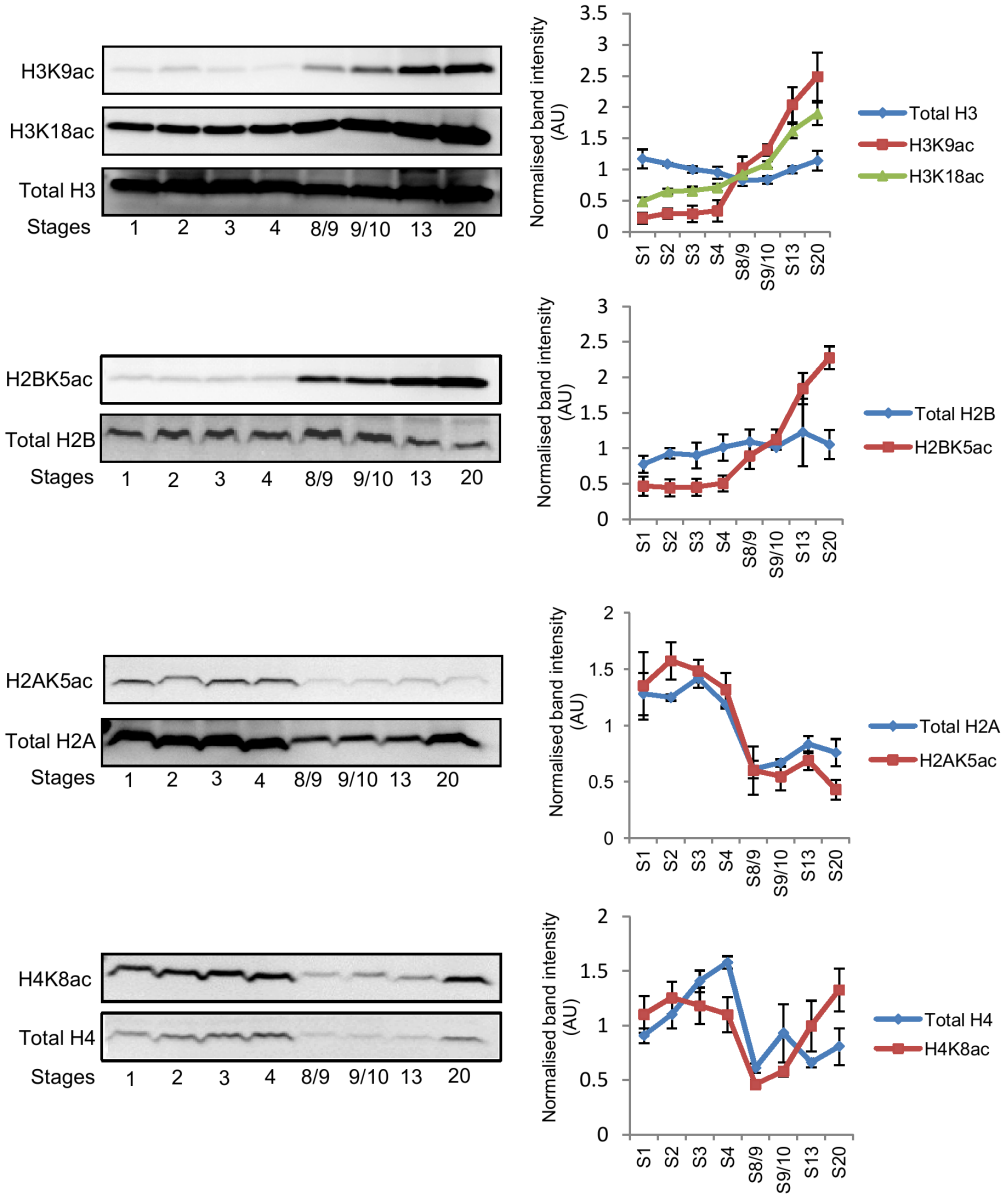
Whole-embryo acetylation levels of core histones during early *Xenopus* embryonic development. 20 embryos collected at different stages of development were lysed in 80 µl lysis buffer and approximately 40 µg of soluble protein was separated by SDS-PAGE, transferred to PVDF membranes and probed with either site-specific anti-acetyl histone antibodies or total anti-histone antibodies. Total and acetylated histones were determined on separate membranes. Representative blots are shown. The graphs show means +/− SEM of normalised band intensity/size obtained from 3 independent fertilisations. The band intensity/size of total and acetylated histones at each stage is expressed relative to the mean of all the stages analysed.

Recent studies in yeast and mammalian cells have suggested that changes in the intracellular level of acetyl CoA can influence protein acetylation [18]–[20]. Therefore, we examined whether there is any correlation between whole-embryo levels of acetyl CoA and general N^ε^-acetylation (simply referred to as acetylation hereafter) of proteins present in whole-embryo lysates. As shown in Figure 4, at the whole-embryo level, bulk acetylation of a number of proteins, as detected by a general anti-acetyl-lysine antibody, appears to correlate with changes in acetyl CoA levels, while acetylation of other proteins are apparently independent of acetyl CoA levels. Changes in the abundance of acetylated proteins during embryonic development shown in Figure 4 may reflect changes in the net rate of acetylation reactions or may simply reflect stage-specific differences in the abundance of individual proteins. Therefore, we decided to look at the acetylation state of specific proteins. The strongest correlation with acetyl CoA levels was observed for protein bands corresponding to approximately 16–18 KDa (Figure 4). These proteins are likely to be histones, which are small proteins of 11–22 KDa containing multiple acetylation sites. Acetylation of histones is developmentally regulated and plays an important role in the control of gene expression during embryogenesis [35], [36]. We therefore examined acetylation of core histones, particularly at stages where an increase in acetyl CoA occurred (stages 8/9–20) (Figure 5). There is no observable change in acetylation of histone H3 K9 and K18 and histone H2B K5 during stages 1–4, but net acetylation of these sites start to increase at stage 8/9, followed by a steady increase until stage 20. The overall pattern is remarkably similar to the pattern of change in acetyl CoA levels observed at these stages. In contrast to histones H3 and H2B, total protein content of both H2A and H4 decreases between stage 4 and stage 8, with little change in net acetylation of H2A K5 and H4K8.

### Microinjection of acetyl CoA into *Xenopus* embryos increases protein acetylation

**Figure 6:**
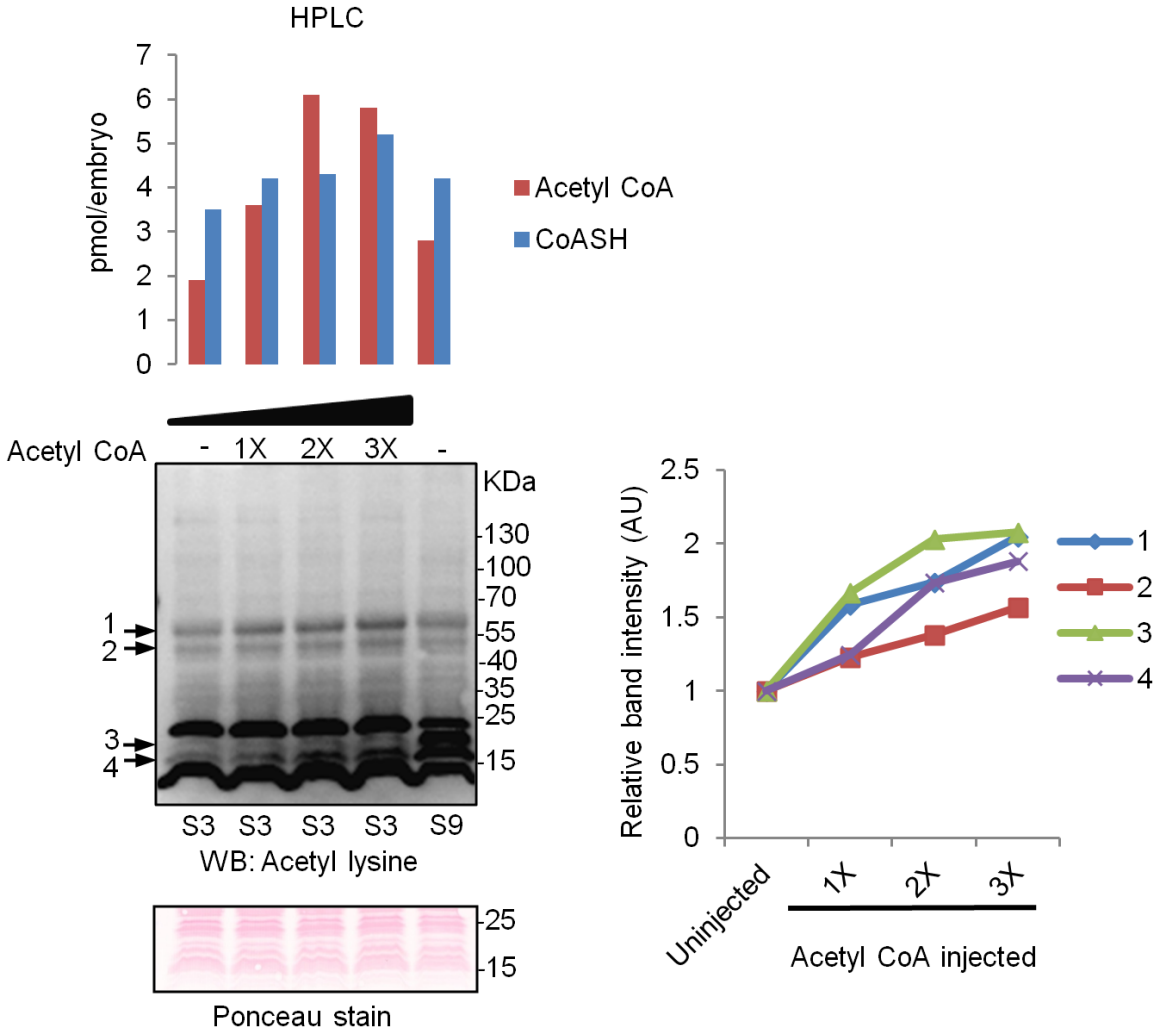
Microinjection of acetyl CoA to stage one embryo increases protein acetylation levels. Immediately after dejellying (30–45 minutes after fertilisation), stage one embryos were microinjected with increasing amounts of acetyl CoA, as described in the Materials and Methods section. Control embryos were injected with water. Injected embryos were allowed to progress to stage 3 and collected for HPLC analysis of CoA compounds or Western blot analysis with anti-acetyl-lysine antibody. For both analyses, 10 injected embryos were used per condition. In addition, embryos were collected after reaching stage 9. Microinjection of acetyl CoA dose dependently increased acetylation levels of several proteins (labelled 1–4). Equal protein loading was confirmed by Ponceau staining of the blot. A section of the Ponceau stained membrane is shown (the complete membrane is shown in Figure S4). For the Western blot shown, linear contrast and brightness adjustment was applied uniformly to the whole blot for clarity. The intensity/size of each of the bands numbered 1–4 was quantified and expressed relative to control embryos.

To examine whether an intracellular increase in acetyl CoA can affect protein acetylation in *Xenopus* embryos, stage one embryos were microinjected with appropriate concentrations of acetyl CoA, so that the resulting increase in acetyl CoA in embryos (up to 3-fold) was within the range of acetyl CoA levels normally observed during embryonic development. The microinjected embryos were allowed to proceed to stage 3 and protein acetylation was examined by Western blot (Figure 6). Microinjection of acetyl CoA dose-dependently increased acetylation of a number of proteins, including proteins whose acetylation normally increases at a later stage (bands labelled 3 and 4 in the blot). This result suggests that a physiological increase in acetyl CoA alone can increase protein acetylation to some extent. However, for proteins labelled 3 and 4 in Figure 6, corresponding to the 16–18 KDa protein bands in Figure 4, even a 3-fold increase in acetyl CoA at stages 1–3 is not enough to bring about the same level of acetylation that is observed at stage 9. In fact, these proteins appear to be heavily acetylated at stage 9, despite the level of acetyl CoA in stage 9 embryos being only about 50% more than that in uninjected stage 3 embryos. This may be due to differences in the expression/activity of the relevant acetyltransferases and/or histone deacetylases between these stages. We also cannot rule out the possibility that microinjection of acetyl CoA increased the expression of these proteins without affecting the net acetylation rate.

## Discussion

In the present study, we have used *Xenopus laevis* as a model organism to measure changes in whole-embryo levels of CoA and acetyl CoA *in vivo* during vertebrate embryonic development. As far as we are aware, the only other study that has measured acetyl CoA levels during the early embryonic development is that by Vastag *et al*, who employed a mass spectrometry based approach to measure changes in 48 common metabolites, including acetyl CoA, during early *Xenopus* embryonic development [37]. Based on their data they concluded that there is no observable change in acetyl CoA levels between fertilisation and 11 h post-fertilisation (corresponding to stage 9). This contradicts our data showing a small but statistically significant increase in acetyl CoA levels between stage 4 and stage 8/9. The discrepancy may be explained by the different approaches used by the two studies for sampling and data analysis. Vastag *et al* measured metabolites in each of 10–11 individual embryos, obtained from three different clutches of eggs, at five different stages between fertilisation and stage 9. Data from individual embryos were then analysed and presented separately. This approach was used to identify metabolites whose concentrations change robustly and consistently in every embryo. Their data did not show a consistent increase in acetyl CoA in every embryo. In our study we pooled 30 embryos for the measurement of CoA species for each stage and combined data from multiple different clutches of embryos for statistical analysis. Our data indicate that statistically, there is a tendency for a small increase in acetyl CoA between stages 4 and 8/9 when a population of embryos is considered. However, such an increase may not be always evident when the level of acetyl CoA in an individual embryo is measured. The average increase in acetyl CoA observed during stage 8/9 was only about 40% of the average level of acetyl CoA during stages 1–4. This may have been obscured by experimental errors associated with measurement. It may also be possible there is a slight embryo-to-embryo and/or clutch-to-clutch variability in the exact timing of the initial increase in acetyl CoA. Such variability may partly arise from the fact that different frogs lay eggs with distinct metabolite concentrations, as observed by Vastag *et al* [37]. Consistent with the points discussed above, closer inspection of the data provided by Vastag *et al* reveals that in two of the three clutches of embryos analysed, approximately half of the embryos actually showed an increase in acetyl CoA levels around 9-11 hours post fertilisation (corresponding to stages 8-9). It must also be noted that in our study a more substantial increase in acetyl CoA levels was observed after stage 9, during stages that were not analysed by Vastag *et al* (between stages 9/10–20).

We have found that acetyl CoA levels continue to increase during stages of gastrulation, neurulation and early organogenesis. While the blastula is mainly composed of undifferentiated cells, during and after gastrulation cells gradually start to diversify and differentiate [28]–[33]. Therefore, whole-embryo levels of CoA and acetyl CoA may not necessarily reflect levels of these compounds in individual cells in an embryo. This may be particularly so after organogenesis, since measurement in adult organisms has shown considerable variations in CoA levels in different tissues, likely reflecting their different metabolic functions [38]. Nevertheless, our data suggest the emergence of cells (entire population or subpopulation of cells in embryos) with increasing levels of acetyl CoA between stages 8/9–13. Recently, a FRET-based probe for retinoic acid has been developed and successfully used in live imaging experiments to study concentration gradients of retinoic acid during embryonic development [39]. Development of such a probe for acetyl CoA would allow us to identify the distribution of acetyl CoA in different groups of cells within an embryo during development.

We have also observed a remarkable similarity between the patterns of changes in acetyl CoA levels during *Xenopus* embryonic development and bulk acetylation levels of a number of proteins, including histones H3 and H2B, at the whole-embryo level. The whole-embryo measurement of acetylated proteins and histones were conducted simply to determine the abundance of the net products of protein acetylation reactions in an embryo as a whole. The whole-embryo levels of acetylated proteins and histones shown in Figures 4 and 5 should not be interpreted as changes in protein acetylation occurring uniformly in all cells in an embryo. Histone acetylation is part of a complex mechanism regulating gene transcription and considerable cell-type specific variations in histone acetylation profile are observed during embryonic development [35], [36]. The apparent correlation between levels of acetyl CoA and protein acetylation levels might be purely coincidental, however a number of recent studies suggest that the level or availability of acetyl CoA is an important determinant for protein acetylation reactions. In yeast and mammalian cells disruption of enzymes that supply nucleocytoplasmic acetyl CoA, ACL and acetyl CoA synthase, is associated with significantly reduced bulk acetylation of histone [21], [40]. Siudeja *et al* showed that chemical inhibition of CoA biosynthesis caused an approximate 50% reduction in total CoA in *Drosophila* cells and a significant reduction in histone and tubulin acetylation [41]. In cultured mammalian cells and in mice nutrient starvation caused a reduction in acetyl CoA and general protein acetylation levels [42]. Conversely, reduced expression of ACC1, a cytosolic enzyme that competes with acetyltransferase for nucleocytoplasmic acetyl CoA, is associated with an increase in bulk histone acetylation in yeast [22]. Tu and co-workers have provided compelling evidence that an increase in intracellular acetyl CoA, during the oxidative phase of the yeast growth cycle, acts as a trigger for initiating transcription of genes involved in growth and cell cycle entry by inducing the Gcn5p/SAGA-catalysed acetylation of histones at growth genes [8], [11]. Notably in their study, levels of acetyl CoA also correlated with global acetylation of histones, including histone H3 K9 and K18, as detected by Western blotting of total cell lysates [8].

In addition to histones and transcriptional factors, it is now known that over 1,700 proteins, including many metabolic enzymes, signalling proteins and other non-DNA binding proteins are targets of acetylation [43]–[46]. This suggests protein acetylation is a post-translational modification as widespread as protein phosphorylation. Changes in levels of acetyl CoA during embryonic development can therefore potentially have wide-ranging downstream effects. It is currently not clear how an increase in intracellular acetyl CoA can affect cellular processes in a specific manner. As shown in Figures 4 and 5, a correlation between the levels of protein acetylation and acetyl CoA was not observed for all proteins. Although we have observed that acetylation of some histone sites appear to increase in tune with acetyl CoA levels after MBT, maternally loaded storage histones are also known to be acetylated. It is likely that protein acetylation, and the degree to which changes in acetyl CoA levels may affect acetylation, still largely depends on the expression/activity of the relevant HATs and HDACs. Close examination of the available literature suggests that the origin of acetyl CoA, and/or subcellular localisation of acetyl CoA, may also confer some level of specificity to the acetylation reactions and downstream processes that an increase in acetyl CoA may affect. In mammalian cells ACL was shown to mediate changes to histone acetylation in response to glucose [21]. Silencing this enzyme reduced histone acetylation without affecting acetylation of tubulin or p53, whereas inhibition of CoA biosynthesis reduced acetylation of both histones and tubulin [21], [41]. Cai *et al* used chromatin immunoprecipitation analysis to show that the increase in Gcn5p/SAGA-mediated histone acetylation, in response to increased acetyl CoA during oxidative phase in yeast, occurred specifically at genes involved in growth [8]. In contrast, disruption of ACC1 in the same organism increased acetylation of histone H3 and H4 in a global untargeted manner [22]. These observations suggest functional coupling of different enzymes that supply acetyl CoA and/or coupling of localised subcellular pools of acetyl CoA with specific HATs and/or subpopulations of HATs.

## Conclusions

In summary, we report the levels of CoA and acetyl CoA during the early embryonic development of *Xenopus laevis*. We observe the levels of acetyl CoA change dramatically during early embryonic development. Such changes in acetyl CoA may potentially play a role in embryonic development by affecting protein acetylation. Future studies should further clarify the causal relationship between acetyl CoA and protein acetylation and its possible functional significance during embryonic development.

## Supporting Information

Figure S1 Identification of CoASH and acetyl CoA peaks using internal standards. HPLC chromatograms showing CoASH and acetyl CoA standards (20 pmol, prepared in water) (a), *Xenopus* stage 8/9 extract (b), and *Xenopus* stage 8/9 extract spiked with 20 pmol CoASH and acetyl CoA internal standards before injection (c). CoASH and acetyl CoA internal standards were added to the neutralised PCA extract before injection. Retention times in minutes: (a) CoASH, 5.22; acetyl CoA, 15.70; (b) CoASH, 5.17; acetyl CoA, 15.51; (c) CoASH, 5.22; acetyl CoA, 15.68. Peak areas: (a) CoASH, 13191.25; acetyl CoA, 13127.75; (b) CoASH, 9337.25; acetyl CoA, 6678; (c) CoASH, 21513.50; acetyl CoA, 18125.50. The retention times of CoASH and acetyl CoA peaks are not affected by the PCA extract. 92% of CoASH and 87% of acetyl CoA standards added to the *Xenopus* PCA extract could be recovered.
(TIF)

Figure S2 The % recovery of CoASH and acetyl CoA standards added during PCA extraction of *Xenopus* embryos. HPLC chromatograms showing CoASH and acetyl CoA standards (50 pmol) (a), *Xenopus* stage 8/9 extract (prepared as described in Materials and Methods) (b), and *Xenopus* stage 8/9 extract in which 200 pmol CoA and acetyl CoA standards were added during the PCA extraction step (c). Retention times in minutes: (a) CoASH, 5.30; acetyl CoA, 15.93; (b) CoASH, 5.29; acetyl CoA, 15.87; (c) CoASH, 5.22; acetyl CoA, 15.61. Peak areas: (a) CoASH, 33972.25; acetyl CoA, 30919; (b) CoASH, 15006; acetyl CoA, 12468.5; (c) CoASH, 50165.5; acetyl CoA, 48421.75. 91% of CoASH and 90% of acetyl CoA standards added to *Xenopus* embryo sample during PCA extraction could be recovered.
(TIF)

Figure S3 Ponceau stained membrane (A) and Coomassie stained gel (B) for the blot presented in Figure 4.
(TIF)

Figure S4 Ponceau stained membrane for the blot presented in Figure 6.
(TIF)

Table S1 Retention times of CoA species for the HPLC analysis. Retention times determined on randomly selected days, spread over a period of 12 months, were used to calculate the mean retention time ± SEM for each compound. The lowest and the highest retention times for each compound, observed over the same time period, are also shown to illustrate the degree of retention time variability.
(DOCX)
