# Supplementary figures and images for: Changes in Acetyl CoA Levels during the Early Embryonic Development of Xenopus laevis
Source: PLoS One. 2014 May 15;9(5):e97693. doi: 10.1371/journal.pone.0097693 (PMC4022644; doi:10.1371/journal.pone.0097693)

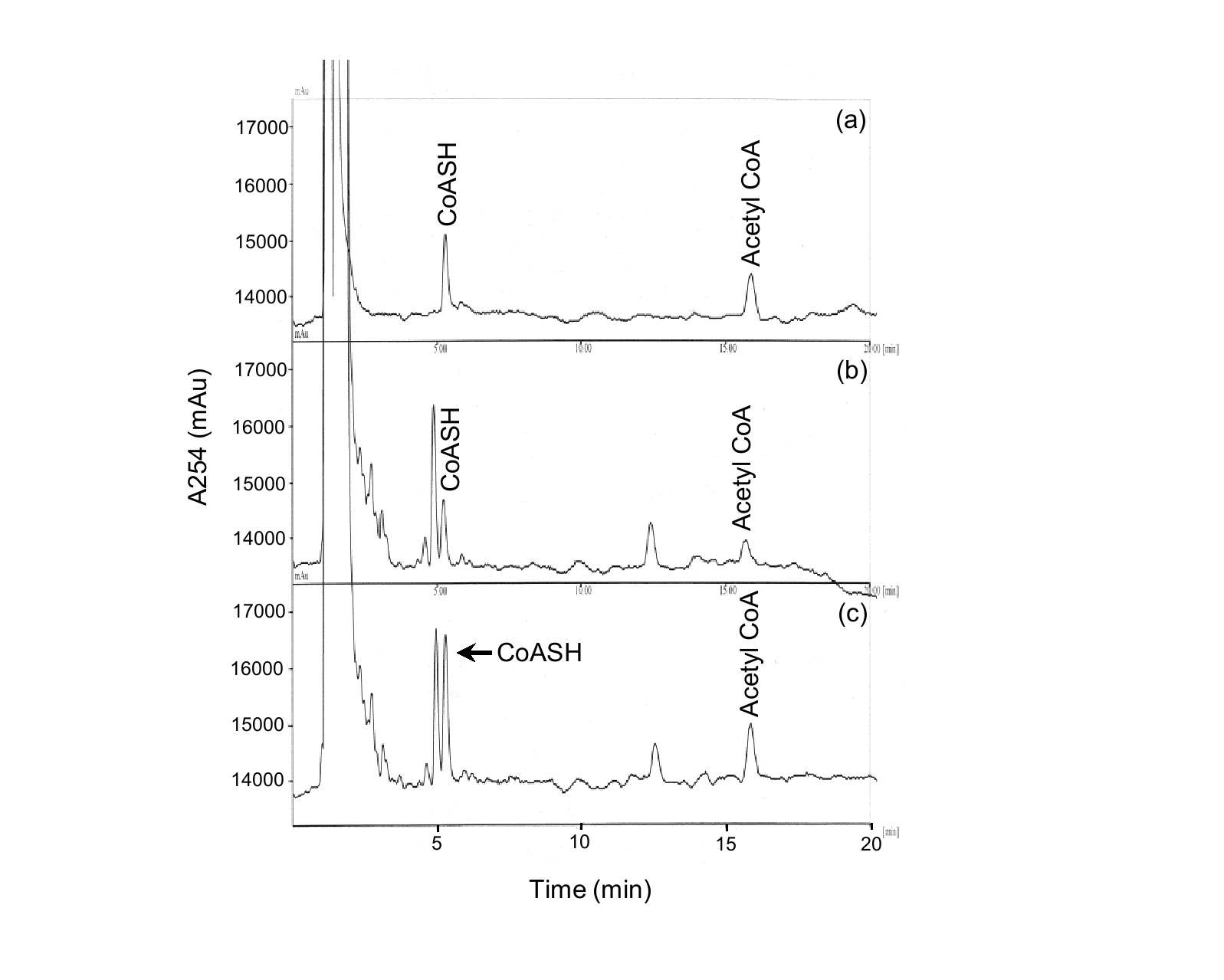

Supplement: Figure S1 — Identification of CoASH and acetyl CoA peaks using internal standards. HPLC chromatograms showing CoASH and acetyl CoA standards (20 pmol, prepared in water) (a), Xenopus stage 8/9 extract (b), and Xenopus stage 8/9 extract spiked with 20 pmol CoASH and acetyl CoA internal standards before injection (c). CoASH and acetyl CoA internal standards were added to the neutralised PCA extract before injection. Retention times in minutes: (a) CoASH, 5.22; acetyl CoA, 15.70; (b) CoASH, 5.17; acetyl CoA, 15.51; (c) CoASH, 5.22; acetyl CoA, 15.68. Peak areas: (a) CoASH, 13191.25; acetyl CoA, 13127.75; (b) CoASH, 9337.25; acetyl CoA, 6678; (c) CoASH, 21513.50; acetyl CoA, 18125.50. The retention times of CoASH and acetyl CoA peaks are not affected by the PCA extract. 92% of CoASH and 87% of acetyl CoA standards added to the Xenopus PCA extract could be recovered. (TIF) [file pone.0097693.s001.tif]

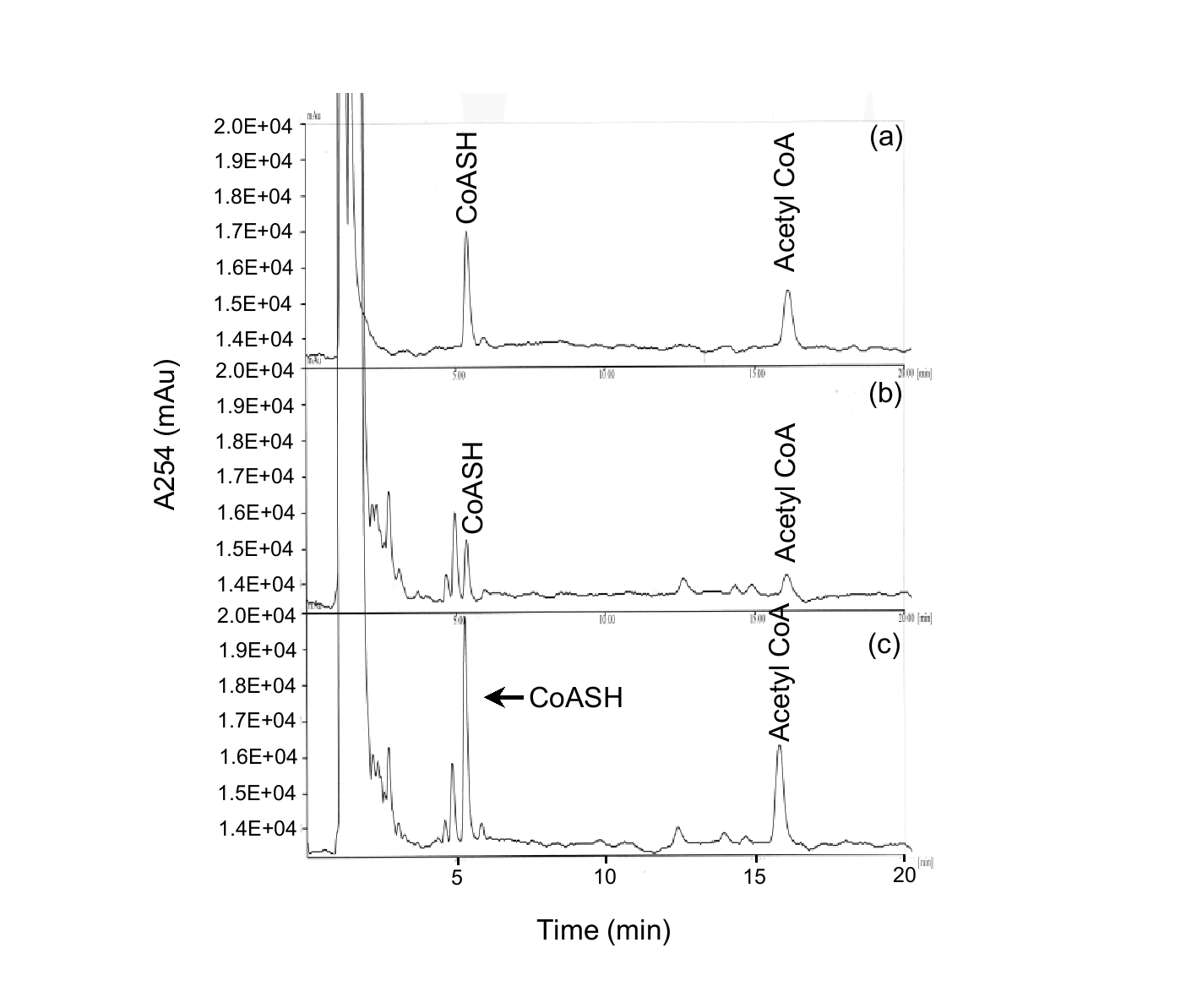

Supplement: Figure S2 — The % recovery of CoASH and acetyl CoA standards added during PCA extraction of Xenopus embryos. HPLC chromatograms showing CoASH and acetyl CoA standards (50 pmol) (a), Xenopus stage 8/9 extract (prepared as described in Materials and Methods) (b), and Xenopus stage 8/9 extract in which 200 pmol CoA and acetyl CoA standards were added during the PCA extraction step (c). Retention times in minutes: (a) CoASH, 5.30; acetyl CoA, 15.93; (b) CoASH, 5.29; acetyl CoA, 15.87; (c) CoASH, 5.22; acetyl CoA, 15.61. Peak areas: (a) CoASH, 33972.25; acetyl CoA, 30919; (b) CoASH, 15006; acetyl CoA, 12468.5; (c) CoASH, 50165.5; acetyl CoA, 48421.75. 91% of CoASH and 90% of acetyl CoA standards added to Xenopus embryo sample during PCA extraction could be recovered. (TIF) [file pone.0097693.s002.tif]

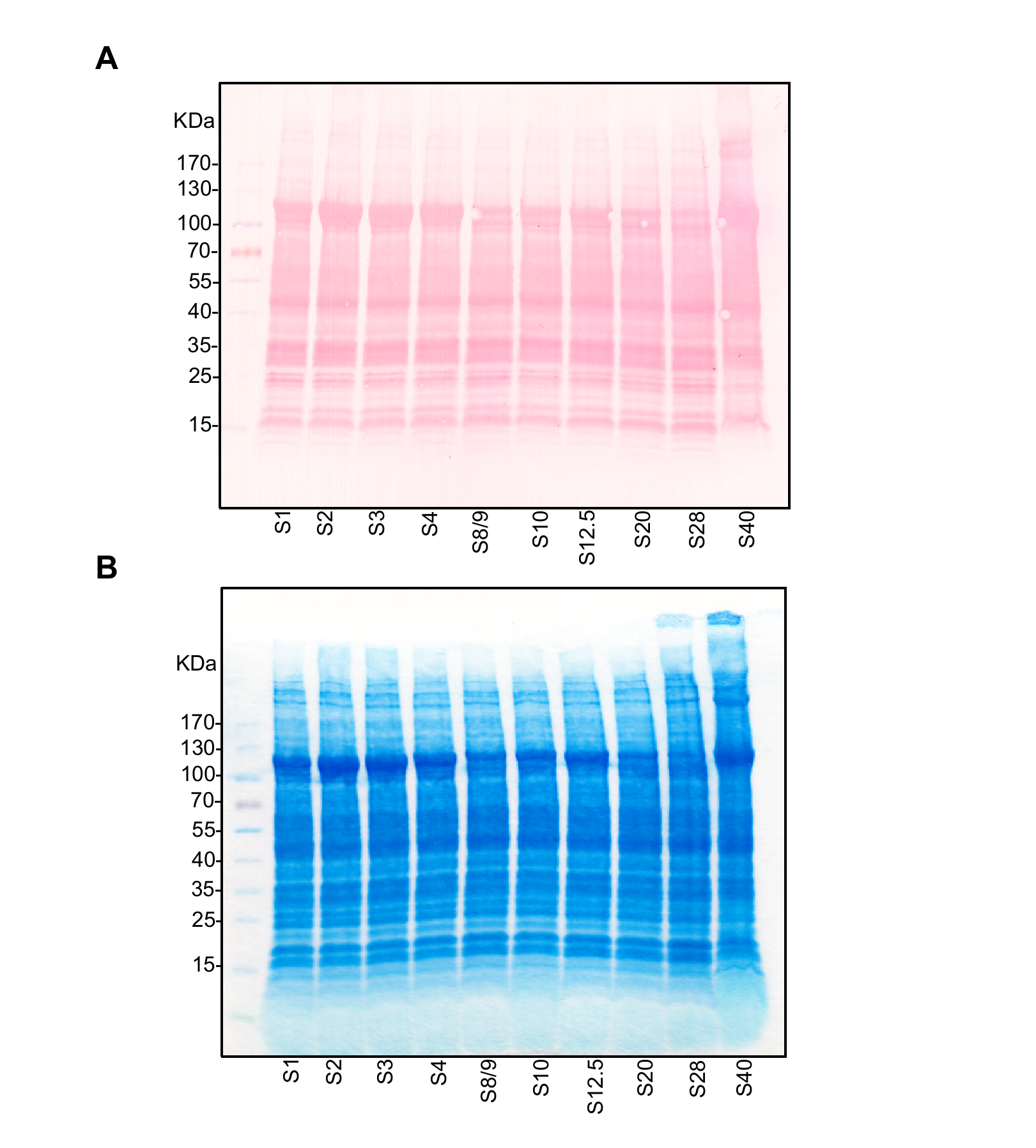

Supplement: Figure S3 — Ponceau stained membrane (A) and Coomassie stained gel (B) for the blot presented in Figure 4. (TIF) [file pone.0097693.s003.tif]

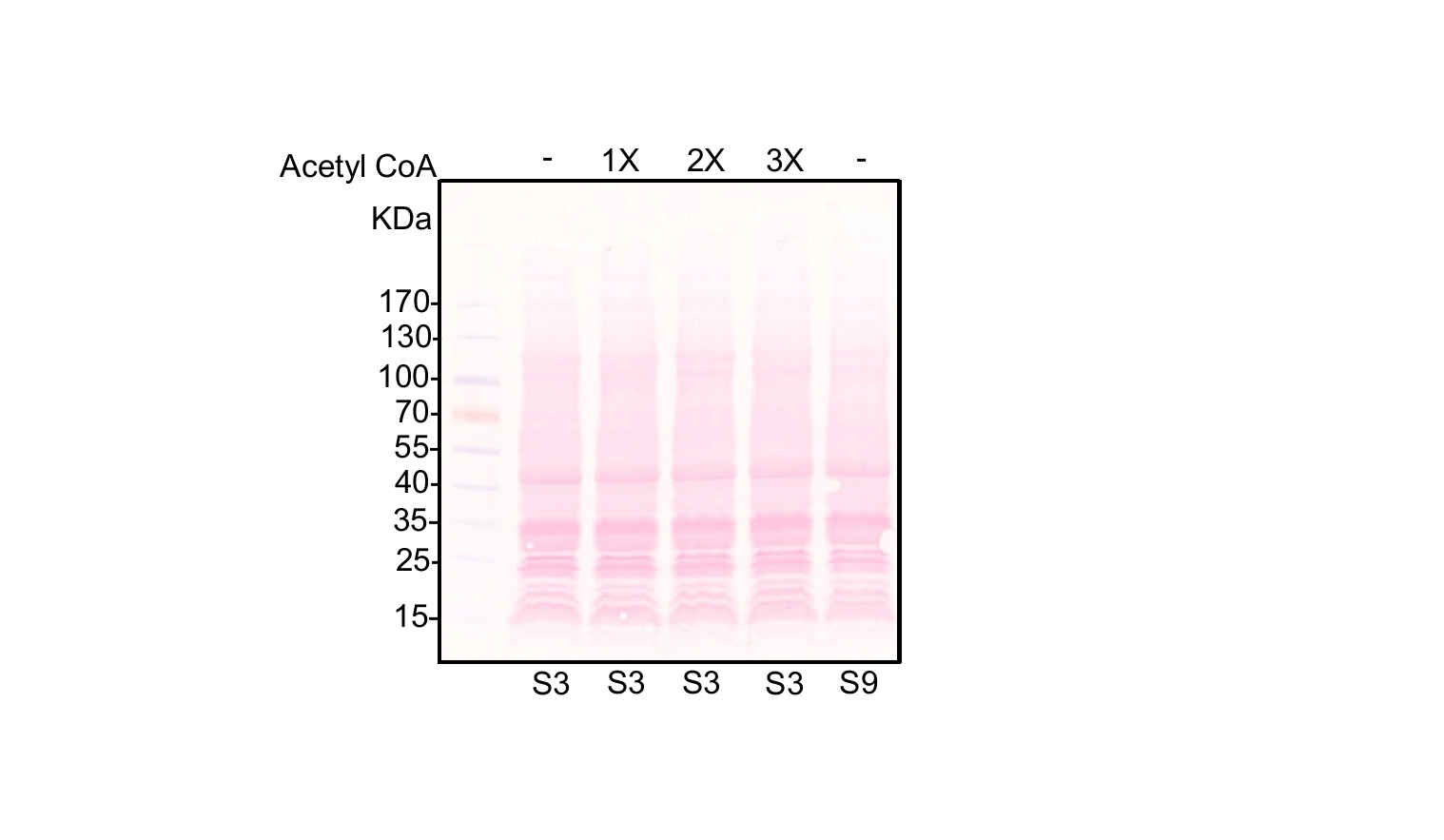

Supplement: Figure S4 — Ponceau stained membrane for the blot presented in Figure 6. (TIF) [file pone.0097693.s004.tif]
