# Supplementary material for: Changes in Acetyl CoA Levels during the Early Embryonic Development of Xenopus laevis
Source: PLoS One. 2014 May 15;9(5):e97693. doi: 10.1371/journal.pone.0097693 (PMC4022644; doi:10.1371/journal.pone.0097693)
Supplement: Table S1 — Retention times of CoA species for the HPLC analysis. Retention times determined on randomly selected days, spread over a period of 12 months, were used to calculate the mean retention time ± SEM for each compound. The lowest and the highest retention times for each compound, observed over the same time period, are also shown to illustrate the degree of retention time variability. (DOCX) [file pone.0097693.s005.docx]

| CoA species | Retention time (min) ± SEM | Range | N |
| --- | --- | --- | --- |
| Malonyl CoA | 3.26 ± 0.05 | 3.16-3.38 | 4 |
| CoASH | 5.18 ± 0.04 | 5.02-5.36 | 9 |
| Methylmalonyl CoA | 7.39 ± 0.08 | 7.26-7.52 | 3 |
| Dephospho CoA | 10.7 ± 0.16 | 10.7-10.93 | 3 |
| Succinyl CoA | 12.23 ± 0.13 | 11.81-12.63 | 7 |
| HMG/acetoacetyl CoA | 14.15 ± 0.18 | 13.47-14.73 | 7 |
| Acetyl CoA | 15.20 ± 0.16 | 14.46-15.81 | 9 |
